# Clinical trial research on COVID-19 in Germany – a systematic analysis

**DOI:** 10.12688/f1000research.55541.1

**Published:** 2021-09-10

**Authors:** Julian Hirt, Abeelan Rasadurai, Matthias Briel, Pascal Düblin, Perrine Janiaud, Lars G. Hemkens

**Affiliations:** 1 International Graduate Academy, Institute for Health and Nursing Science, Medical Faculty, Martin Luther University Halle-Wittenberg, Magdeburger Strasse 8, 06112 Halle (Saale), Germany; 2 Department of Clinical Research, University Hospital Basel, University of Basel, Basel, Spitalstrasse 12, 4031 Basel, Switzerland; 3 Department of Health Sciences and Technology, ETH Zürich, Universitätstrasse 2, 8092 Zurich, Switzerland; 4 Department of Health Research Methods, Evidence, and Impact, McMaster University, 1280 Main Street West, Hamilton L8S 4L8, Ontario, Canada; 5 Meta-Research Innovation Center Berlin (METRIC-B), Berlin Institute of Health, Anna-Louisa-Karsch-Strasse 2, 10178 Berlin, Germany; 6 Meta-Research Innovation Center at Stanford (METRICS), Stanford University, 1265 Welch Road, Stanford, California 94305, USA

**Keywords:** COVID-19, SARS-CoV-2, Randomized clinical trials, Germany

## Abstract

**Background:** In 2020, the COVID-19 pandemic led to an unprecedented volume of almost 3,000 clinical trials registered worldwide. We aimed to describe the COVID-19 clinical trial research agenda in Germany during the first year of the pandemic. **Methods:** We identified randomized clinical trials assessing interventions to treat or prevent COVID-19 that were registered in 2020 and recruited or planned to recruit participants in Germany. We requested recruitment information from trial investigators as of April 2021. **Results:** In 2020, 65 trials were completely (n=27) or partially (n=38) conducted in Germany. Most trials investigated interventions to treat COVID-19 (86.2%; 56/65), in hospitalized patients (67.7%; 44/65), with industry funding (53.8%; 35/65). Few trials were completed (21.5%; 14/65). Overall, 187,179 participants were planned to be recruited (20,696 in Germany), with a median number of 106 German participants per trial (IQR 40 to 345). From the planned German participants, 13.4% were recruited (median 15 per trial (IQR 0 to 44). **Conclusions:** The overall German contribution to the worldwide COVID-19 clinical trial research agenda was modest. Few trials delivered urgently needed evidence. Most trials did not meet recruitment goals. Evaluation and international comparison of the challenges for conducting clinical trials in Germany is needed.

## Introduction

The COVID-19 pandemic has led to an unprecedented volume and speed in the international clinical research agenda. Almost 700 clinical trials were registered worldwide in the first 100 days, which planned to recruit almost 400,000 participants to assess interventions to treat or prevent COVID-19. ^1^ However, this research agenda has been tainted by a multitude of small trials with 50 from the 700 trials aimed to include more than 1,000 participants. ^1^ Nevertheless, there were remarkable efforts to conduct large adaptive pragmatic trials directly informing therapeutic decisions in COVID-19 patients: the UK Randomised Evaluation of COVid-19 thERapY (RECOVERY) trial recruited 10,000 patients in two months, ^2^ the established Randomised, Embedded, Multifactorial Adaptive Platform trial for Community-Acquired Pneumonia (REMAP-CAP) was rapidly adapted to COVID-19, ^3^ and the COVID-19 Solidarity Trial for COVID-19 Treatments (SOLIDARITY) sponsored by the World Health Organization (WHO), was conducted in six months. ^4^ In the first 100 days, China, the US, Spain and France, had the largest share of the initiated trials, with many planned as international collaborations. ^1^ Germany contributed to large-scale international studies such as REMAP-CAP ^5^ and WHO-SOLIDARITY, ^6^ and the German Federal Ministry for Education and Research (BMBF) provided 1.6 billion € for COVID-19-related research. ^7^

The aim of this study was to describe the German contribution to the worldwide COVID-19 clinical trial agenda, including all randomized clinical trials (RCTs) with German participants, that were registered during the first year of the pandemic.

## Methods

### Data sources

We searched the COVID-evidence database to identify RCTs in international trial registries ( ClinicalTrials.gov; WHO International Clinical Trials Registry Platform). Additionally, we manually searched the German Clinical Trials Register (DRKS). For all eligible registered trials, we searched corresponding publication results and preprints in the Living OVerview of Evidence platform for COVID-19 (L·OVE), Cochrane COVID-19 Study Register, MEDLINE/PubMed, and Google Scholar using the trial registry number (last search 1 April 2021, see extended data ^8^).

### Eligibility criteria

We included all planned, ongoing, or completed RCTs assessing interventions to treat or prevent COVID-19, registered in 2020, that recruited or planned to recruit at least one participant in Germany.

### Selection of trials and data extraction

One author (JH) conducted the searches and screened the trials for eligibility and a second author (PJ or LGH) was consulted in cases where eligibility was unclear. Four authors (JH, PJ, AR, MB) extracted data on trial characteristics (status, duration, design, population, intervention and control, recruiting countries, actual/planned trial size, funding) and published results.

Trials with industrial/commercial sponsors were classified as industry-funded, those with public/non-commercial sponsors as public-funded, and trials with public/non-commercial sponsors with collaborators from industry as publicly funded with industry contribution. Trials were also classified as completely (i.e., national trials) or partially (i.e., international) conducted in Germany. Details on the extraction process are provided as extended data. ^8^

### Recruitment information

We identified contact details of corresponding investigators for all eligible trials using registry information and web search. For each trial, we asked investigators via email about the current trial status, inclusion date of the first patient, number of patients recruited in Germany, and any published results (prior to April 2021, see extended data ^8^). For trials completely conducted in Germany with public funding, we further asked (i) if they were aware of other trials with public funding that we have not identified and (ii) to confirm the extracted information on their trial (see extended data ^8^). We received replies from 60% (39 out of 65) of all eligible trials. This complemented the data contained in registries or publications and provided full information on actual and target sample size in Germany for 34 trials (52.3%).

### COVID-evidence update

COVID-evidence is a living database that is continuously being updated. ^9, 10^ Registry entries retrieved weekly from ClinicalTrials.gov and the WHO International Clinical Trials Registry Platform are automatically pre-screened for eligibility using basic filters to identify RCTs. Unclear and identified RCT entries are then manually screened to verify eligibility.

In August 2021, the RCT filter of COVID-evidence was updated. The data presented herein are based on the COVID-evidence processes (including RCT filter and automatic pre-screening) as of April 2021. For transparency and exhaustiveness, we updated our search in the COVID-evidence database in August 2021 with the use of the most recent processes and post-hoc identified trials.

### Data analysis

We report medians with interquartile ranges (IQRs) if not stated otherwise. For all analyses, we used R (version 4.1).

## Results

In 2020, 65 RCTs were planned to investigate treatments or preventive interventions for COVID-19 with participants from Germany ( Table 1 and extended data ^8^). They aimed to include a median of 300 participants per trial (IQR, 174 to 830) internationally, including 106 in Germany (IQR, 40 to 345, Table 2).

**Table 1.** Characteristics of all 65 randomized COVID-19 trials planned to be conducted in Germany.

	Total	Tested intervention		Funding*		Partially conducted in Germany	Completely conducted in Germany
		Treatment	Prevention	Industry funding	Public funding		
	n = 65	n = 56	n = 9	n = 35	n = 25	n = 38	n = 27
**Intervention type**							
Device or procedure (e.g. ECMO)	7 (10.8%)	7 (12.5%)	0 (0%)	1 (2.9%)	6 (24%)	1 (2.6%)	6 (22.2%)
Drug or biological	48 (73.8%)	48 (85.7%)	0 (0%)	26 (74.3%)	18 (72%)	30 (78.9%)	18 (66.7%)
Multiple**	1 (1.5%)	1 (1.8%)	0 (0%)	0 (0%)	1 (4%)	1 (2.6%)	0 (0%)
Vaccine	9 (13.8%)	0 (0%)	9 (100%)	8 (22.9%)	0 (0%)	6 (15.8%)	3 (11.1%)
**Control type**							
Placebo	37 (56.9%)	28 (50%)	9 (100%)	25 (71.4%)	9 (36%)	24 (63.2%)	13 (48.1%)
Usual care	26 (40%)	26 (46.4%)	0 (0%)	10 (28.6%)	15 (60%)	14 (36.8%)	12 (44.4%)
Multiple***	2 (3.1%)	2 (3.6%)	0 (0%)	0 (0%)	1 (4%)	0 (0%)	2 (7.4%)
**Setting**							
Inpatients	44 (67.7%)	44 (78.6%)	0 (0%)	24 (68.6%)	18 (72%)	28 (73.7%)	16 (59.3%)
Inpatients and outpatients	7 (10.8%)	7 (12.5%)	0 (0%)	2 (5.7%)	3 (12%)	3 (7.9%)	4 (14.8%)
Outpatients	12 (18.5%)	5 (8.9%)	7 (77.8%)	7 (20%)	4 (16%)	7 (18.4%)	5 (18.5%)
Healthcare workers	2 (3.1%)	0 (0%)	2 (22.2%)	2 (5.7%)	0 (0%)	0 (0%)	2 (7.4%)
**Blinding**							
Double-blind	36 (55.4%)	28 (50%)	8 (88.9%)	22 (62.9%)	10 (40%)	22 (57.9%)	14 (51.9%)
Single-blind	3 (4.6%)	2 (3.6%)	1 (11.1%)	3 (8.6%)	0 (0%)	2 (5.3%)	1 (3.7%)
Open-label	26 (40%)	26 (46.4%)	0 (0%)	10 (28.6%)	15 (60%)	14 (36.8%)	12 (44.4%)
**Status**							
Ongoing	35 (53.8%)	27 (48.2%)	8 (88.9%)	17 (48.6%)	13 (52%)	18 (47.4%)	17 (63%)
Not yet recruiting	6 (9.2%)	5 (8.9%)	1 (11.1%)	3 (8.6%)	3 (12%)	4 (10.5%)	2 (7.4%)
Terminated	9 (13.8%)	9 (16.1%)	0 (0%)	5 (14.3%)	4 (16%)	5 (13.2%)	4 (14.8%)
Completed	14 (21.5%)	14 (25%)	0 (0%)	10 (28.6%)	4 (16%)	11 (28.9%)	3 (11.1%)
Withdrawn	1 (1.5%)	1 (1.8%)	0 (0%)	0 (0%)	1 (4%)	0 (0%)	1 (3.7%)
**Published results**							
Yes	17 (26.2%)	15 (26.8%)	2 (22.2%)	12 (34.3%)	4 (16%)	15 (39.5%)	2 (7.4%)
No	48 (73.8%)	41 (73.2%)	7 (77.8%)	23 (65.7%)	21 (84%)	23 (60.5%)	25 (92.6%)

Abbreviations: ECMO = extracorporeal membrane oxygenation; n = number.

Notes: * Five trials had public funding with industry contribution, details are provided in eTable 1a ( extended data ^8^); ** One trial used a drug or biological substance or procedure as intervention type; *** Two trials used a placebo or usual care as control type.

**Table 2.** Number of planned and actually recruited participants overall and from Germany in randomized COVID-19 trials.

	Total (n=65)		Tested intervention			
			Treatment (n=56)	N Trials*	Prevention (n=9)	N Trials*
	Median [IQR; range] N Participants	N Trials *	Median [IQR; range] N Participants (all trials)		Median [IQR; range] N Participants (all trials)	
**Planned to be recruited overall** *(internationally and in Germany)*	300 [174 to 830, 24 to 46,663] 187,179	62	256 [172 to 450, 24 to 11,266] 33,540	53	2,520 [1,200 to 34,000, 168 to 46,663] 153,639	9
**Planned to be recruited in Germany**	106 [40 to 345, 1 to 3,000] 20,696	55	80 [38 to 205, 1 to 2,700] 10,613	48	1,200 [550 to 2,279, 225 to 3,000] 10,083	7
**Actually recruited in Germany**	15 [0 to 44, 0 to 516] 1,579	38	14 [0 to 40, 0 to 516] 1,373	35	59 [29 to 103, 0 to 147] 206	3
**Median proportion of target sample size** *(actual Germany/planned Germany)*	13.4% ** [0 to 29.2%, 0 to 113%]	34	13.5% [0 to 29.1%, 0 to 113%]	32	2.5% [1.2% to 3.7%, 0 to 4.9%]	2
Abbreviations: IQR = interquartile range; N/n = number. Notes: * The number of trials corresponds to trials with full recruitment information; ** For the 13 trials that provided recruitment information and announced a completion date by April 2021 (timepoint of the recruitment status assessment), the median proportion of target sample size was 13.6% (IQR, 0.6 to 24.0%, range 0 to 113%).						

	Funding**			N Trials*	Partially conducted in Germany (n=38)	N Trials*	Completely conducted in Germany (n=27)	N Trials*
	Industry funding (n=35)	Public funding (n=25)						
	Median [IQR; range] N Participants	N Trials*	Median [IQR; range] N Participants		Median [IQR; range] N Participants		Median [IQR; range] N Participants	
**Planned to be recruited overall** *(internationally and in Germany)*	3,784 [200 to 1,158, 40 to 46,663] 128,666	34	229 [104 to 649, 24 to 11,266] 21,258	24	402 [210 to 967, 40 to 46,663] 172,782	35	200 [103 to 500, 24 to 2,700] 14,397	27
**Planned to be recruited in Germany**	50 [29 to 200, 1 to 2,520] 8,328	28	200 [90 to 374, 20 to 2,700] 8,613	23	50 [26 to 200, 1 to 3,000] 6,299	28	200 [103 to 500, 24 to 2,700] 14,397	27
**Actually recruited in Germany**	5 [0 to 31, 0 to 147] 319	14	19 [10 to 45, 0 to 516] 1,106	21	5 [0 to 13%, 0 to 147%] 328	18	31 [15 to 61, 0 to 516] 1,251	20
**Median proportion of target sample size** *(actual Germany/planned Germany)*	2.5% [0 to 18.1%, 0 to 28%]	12	13.5% [1.3% to 52.9%, 0 to 113%]	20	6.5% [0 to 18.4%, 0 to 50%]	14	14.9% [4.1% to 52.9%, 0 to 113%)	20
Abbreviations: IQR = interquartile range; N = number. Notes: * The number of trials corresponds to trials with full recruitment information; ** Five trials had public funding with industry contribution and are not considered in this table.								

After a peak with 18 registrations in April 2020, one to nine trials were registered each month. As of 21 May 2021, 35 were ongoing (53.8%), six not yet recruiting (9.2%), nine terminated early (13.8%), 14 (21.5%) completed, and one withdrew and will never recruit (1.5%).

Our results also indicate that 17 trials (26.2%) had published results; 15 were partially conducted in Germany, 15 were explored COVID-19 therapies and 12 were industry-funded. From those 17 trials with published results, 12 trials had results published as peer-reviewed articles or preprints, and five trials had published results exclusively as press release or in the registry. Results were reported by 11 trials, while six stated interim results ( Table 3).

**Table 3.** Trial results summary and recruitment status of all 17 trials with published results.

Trial acronym	Summary	Actual/target sample size
**GS-US-540-5773** (NCT04292899)	Remdesivir for 5 versus 10-days in hospitalized patients with severe Covid-19 not requiring mechanical ventilation on clinical status at day 14, assessed on a 7-point ordinal scale. No stat. significant difference between a 5-days and a 10-days course (65% and 54% patients, respectively, showed a clinical improvement of at least 2 points (p=0.14)). Results are based on 402 randomized patients. The trial was then extended to include additional patients. Results reported in a peer reviewed publication ^24^ and posted on Clinicaltrials.gov (NCT04292899); industry-funded.	International: 4,838/n.r. Germany: 43/n.r.
**GS-US-540-5774** (NCT04292730)	Remdesivir for 5 days or 10 days vs standard care in patients with moderate COVID-19 on clinical status at day 11, assessed on a 7-point ordinal scale. Patients randomized to 5 days remdesivir had a stat. significant benefit (odds ratio, 1.65, 95% CI [1.09; 2.48], p = 0.02) but not those randomized to 10 days remdesivir (p=0.18). Results are based on 596 randomized patients. The trial was then extended to include additional patients. Results reported in a peer reviewed publication ^25^ and posted on Clinicaltrials.gov (NCT04292730) and Clinicaltrialsregister.eu (EUCTR2020-000842-32-DE); industry-funded.	International: 1,087/1,600 Germany: 37/200
**ACTT-1** (NCT04280705)	Remdesivir versus placebo in hospitalized patients with COVID-19 with evidence of lower respiratory tract infection on the time to recovery, defined by either discharge from the hospital or hospitalization for infection-control purposes only assessed on an 8-point ordinal scale. Remdesivir had a stat. significant benefit compared with placebo (rate ratio for recovery, 1.29, 95% CI [1.12; 1.49], p < 0.001). Results reported in a peer reviewed publication ^26^ and posted on Clinicaltrialsregister.eu (EUCTR2020-001052-18-DE); industry-funded.	International: 1,062/800 Germany: 13/100
**PANAMO** (NCT04333420)	A phase 2/3 trial on anti-C5a antibody IFX-1 (vilobelimab) versus standard care in hospitalized patients with severe COVID-19 pneumonia on percentage change in PaO _2_/FiO _2_ in the supine position from baseline to day 5. Results focus on the phase 2 which randomized 30 patients in the Netherlands. No stat. significant difference for the percentage change in PaO _2_/FiO _2_ (difference -24%, 95% CI [−58; 9], p = 0.15), but preliminary findings on mortality at 28-days promising enough to continue with phase 3 and mortality as primary endpoint. Results reported in a peer reviewed publication ^27^; industry-funded.	International: n.r./390 Germany: n.r./40
**COVACTA** (NCT04320615)	Tocilizumab versus standard of care in hospitalized patients with severe COVID-19 pneumonia on clinical status at day 28, assessed on a 7-point ordinal scale. No stat. significant difference for clinical status (between-group difference −1.0, 95% CI [−2.5; 0]; p = 0.31). Results reported in a peer reviewed publication ^28^; public and industry funding.	International: 452/450 Germany: n.r./50
**NCT04327388**	Sarilumab (200 mg or 400 mg) versus placebo in hospitalized patients receiving supplemental oxygen with severe or critical COVID-19 on time to clinical improvement of two or more points on a 7-point ordinal scale. No stat. significant difference for time to clinical improvement (200 mg: HR 1.03, 95% CI [0.75; 1.4]; p = 0.96 and 400 mg: hazard ratio 1.14, 95% CI [0.84; 1.54], p = 0.34). Results reported in a peer reviewed publication ^29^ and posted on Clinicaltrials.gov (NCT04327388); industry-funded.	International: 420/440 Germany: 6/25
**REMAP-CAP** (NCT02735707)	Adaptive platform trial assessing multiple interventions in hospitalized patients with severe COVID-19 on an ordinal scale combining organ support-free days and in-hospital deaths at day 21. No stat. significant benefit on mortality for hydroxychloroquine (OR 1.04,95% CI [0.49; 2.18]) assessed on 142 patients randomized. ^30^ Treatment with a 7-day fixed-dose course of hydrocortisone or shock-dependent dosing of hydrocortisone suggested a benefit assessed on 384 patients randomized. The intervention arms were terminated early following the press release from the RECOVERY trial showing a stat. significant benefit on mortality outcomes. ^31^ Both tocilizumab and sarilumab (interleukin-6 receptor antagonists) met the predefined criteria for efficacy assessed on 803 patients randomized. ^32^ Therapeutic anticoagulation did not improve hospital survival or days free of organ support compared to usual care pharmacological thromboprophylaxis assessed on 1,074 patients randomized. The intervention arm was stopped for futility. ^33^ The convalescent plasma intervention arm reached a pre-specified statistical threshold for futility among patients who are critically ill with COVID-19. Although recruitment continued for moderate patients, following the publication of the RECOVERY trial results showing no stat. significant benefit on mortality outcomes the convalescent plasma intervention arm was closed to recruitment. ^34, 35^ Results reported in multiple peer reviewed publications, ^30– 32^ preprints ^33^ and press releases ^34, 35^; industry-funded.	International: 11,557/n.r. * Germany: n.r./n.r.
**EUCTR2020-001270-29-DE**	A phase 2/3 adaptive trial assessing hydroxychloroquine in hospitalized patients with moderate to severe COVID-19 on changes from baseline in oxygen saturation/fraction of inspired oxygen ratio at day 15. The trial was terminated early due to enrollment and feasibility challenges, and due to evidence suggesting no benefit from hydroxychloroquine. No statistical analysis was conducted due to the early termination of the trial. Results posted on Clinicaltrialsregister.eu (EUCTR2020-001270-29-DE); industry-funded.	International: 14/350 Germany: 0/80
**RUXCOVID** (NCT04362137)	Ruxolitinib versus placebo in patients aged ≥12 years hospitalized for COVID-19 and not intubated or receiving intensive care prior to randomization on the proportion of patients who die, develop respiratory failure [require mechanical ventilation] or require intensive care by day 29. No stat. significant benefit for ruxolitinib. Results communicated in a press release ^36^ and posted on Clinicaltrialsregister.eu (EUCTR2020-001662-11-DE); industry-funded.	International: 432/402 Germany: 9/50
**NCT04368728**	Phase 1/2/3 assessing the safety, tolerability, immunogenicity, and efficacy of RNA Vaccine Candidates (BNT162b2 - Pfizer–BioNTech) against COVID-19. The ongoing phase 3 preliminary results showed that a two-dose regimen of BNT162b2 conferred 95% protection against Covid-19 in persons 16 years of age or older and the safety over a median of 2 months was similar to that of other viral vaccines. Results reported in multiple peer reviewed publications ^37– 39^; industry-funded.	International: 43,548/46,663 Germany: n.r./500
**Mir-Age** (NCT04393038)	ABX464 versus placebo to treat inflammation and prevent acute respiratory failure in high-risk patients with COVID-19 on the rate of patients with no invasive or non-invasive mechanical ventilation. Terminated early following the Data Safety and Monitoring Board recommendation for lack of efficacy based on the interim analysis of 305 high-risk Covid-19 patients who completed the study period. No quantitative data were reported. Results communicated in a press release ^40^; industry-funded.	International: 500/1,034 Germany: n.r./200
**CAN-COVID** (NCT04382053)	Canakinumab versus placebo in hospitalized patients with COVID-19 pneumonia and cytokine release syndrome on survival without the need for mechanical ventilation at day 29. No stat. significant benefit was shown for canakinumab (88.8% responders) versus placebo (85.7% responders, p = 0.29). Results communicated in a press release ^41^; industry-funded.	International: 143/120 Germany: n.r./50
**COV-BARRIER** (NCT04421027)	Baricitinib versus placebo in hospitalized patients with COVID-19 on the composite endpoint percentage of participants who die or require non-invasive ventilation/high-flow oxygen or invasive mechanical ventilation at 28 days. No stat. significance for the primary endpoint (OR 0.85, 95% CI [0.67; 1.08]) but data showed 38% reduction in mortality by day 28 (p=0.0018) in patients treated with baricitinib in addition to standard of care including corticosteroids and remdesivir. Results communicated in a press release ^42^; industry-funded.	International: 1525/1400 Germany: n.r./30
**CureVac AG** (NCT04449276)	Safety and immunogenicity profile after 1 and 2 dose administrations of CVnCoV-vaccine at different dose levels in healthy adults. Interim analysis of an ongoing phase 1 trial showed that two doses of CVnCoV ranging from 2 μg to 12 μg per dose, administered 28 days apart were safe and were able to boost the pre-existing immune response even at low dose levels. This study allowed to select the 12 μg dose for further clinical investigation, including a phase 2b/3 study investigating efficacy, safety, and immunogenicity of the candidate vaccine CVnCoV. Results reported in a preprint ^43^; industry-funded.	International: 248/168 Germany: n.r./n.r.
**ACTIV-3/TICO** (EUCTR2020-003278-37-DK)	A platform adaptive trial assessing multiple interventions versus placebo in hospitalized patients with COVID-19 without end-organ failure on sustained recovery during a 90-day period. The results reported on 314 patients focus on the neutralizing monoclonal antibody LY-CoV555 when co-administered with remdesivir which did not demonstrate stat. significant efficacy (OR 0.85, 95% CI [0.56; 1.29], p=0.45). This intervention arm was terminated early for futility following the data and safety monitoring board recommendation, but the trial is ongoing. Results reported in a peer review publication ^44^; industry-funded.	International: n.r./n.r. * Germany: 0/n.r.
**CYCOV** (NCT04324528)	Cytokine removal therapy in inpatients requiring veno-venous ECMO on interleukin-6 level on serum interleukin-6 (IL-6) concentration 72 h after initiation of ECMO. Adjusted mean log IL-6 concentrations after 72 h were 0.30 higher in the cytokine adsorption group (95% CI -0.70 to 1.30, p=0.54). In addition, early initiation of cytokine adsorption had a negative effect on survival. Results reported in a peer review publication ^45^; industry-funded.	International: n.a. Germany 34/30
**CAPSID** (EUCTR2020-001310-38-DE)	Convalescent plasma in inpatients on a composite endpoint of survival and no longer fulfilling criteria of severe COVID-19. There was no stat. significant difference with 43.4% of patients in the convalescent plasma group and 32.7% in the control group reaching the primary outcome (p=0.32). A pre-defined subgroup analysis showed a significant benefit for convalescent plasma among those who received a larger amount of neutralizing antibodies. Results posted as a preprint ^46^; industry-funded.	International: n.a. Germany: 105/106

Abbreviation: ECMO = extracorporeal membrane oxygenation; n.a. = not applicable; n.r. = not reported; stat. = statistically; CI = confidence interval.

Notes: * Adaptive designs, the target sample size is continuously re-evaluated.

### Topic and design

From the 65 trials, 56 investigated COVID-19 therapies (86.2%) with a total planned sample size of 33,540 participants internationally (median 256 per trial [IQR, 172 to 450]); seven (12.5%) planned to recruit more than 1,000 participants.

Drugs and biologicals (e.g., convalescent plasma) were investigated in 48 of 65 trials (73.8%). The spectrum of treatments was wide, including antivirals (n = 10, 20.8%, e.g., remdesivir n = 4), monoclonal antibodies (n = 8, 16.7%, e.g., tocilizumab n = 3), convalescent plasma (n = 6, 2.5%), hydroxychloroquine (n = 5, 10.4%), and kinase inhibitors (n = 4, 8.3%, e.g., baricitinib n = 1).

All trials assessing interventions to prevent COVID-19 were vaccine trials (13.8%; 9 from 65). They were considerably larger than therapy trials with a total planned sample size of 153,639 participants (median 2,520 per trial [IQR, 1,200 to 34,000]); seven of the nine vaccine trials (77.7%) planned to include over 1,000 participants. From the 187,179 planned participants in the 65 trials, 82.1% were healthy participants planned to be recruited in vaccine trials.

None of the trials investigated non-pharmaceutical interventions to prevent the pandemic spread, such as social distancing or behavioral interventions.

Hospitalized patients were recruited in 44 (67.7%), outpatients in 14 (21.5%), and both inpatients and outpatients in seven (10.8%) trials. No trial was conducted in nursing homes, kindergarten, childcare, or schools ( Table 1 and extended data ^8^). Adolescents (12 years and older) were included in five trials partially conducted in Germany, however, trials that included children below the age of 12 did not exist.

Most trials were double blinded (n = 36, 55.4%) and used a two-arm parallel group design (n = 54, 83.1%). From the 11 trials that had an adaptive design (17.0%), five re-estimated the target sample size.

### Funding and internationality

From the 65 trials, 35 (53.8%) were industry-funded, 25 (38.5%) publicly funded, and five (7.7%) publicly funded with industry contribution. None of the publicly funded trials assessed a vaccine ( Table 4). In these 65 trials, 38 were partially conducted in Germany (58.5%), which planned to recruit 172,782 participants across all included countries (median 402 per trial [IQR, 210 to 967]). Additionally, there were 27 trials completely conducted in Germany (41.5%) that planned to recruit 14,397 participants (median 200 per trial [IQR, 103 to 500]) in 125 German study centers (median 1.5 centers per trial [IQR, 1 to 8.8]).

**Table 4.** Characteristics of all 22 randomized COVID-19 trials planned to be conducted in Germany that received public funding.

Trial acronym (ID)	Topic (blinding)	Target sample size	Start to end date	Trial center(s)	Published results
**Ongoing trials (n = 12 trials, actually recruited a median proportion of 27.3% of the target sample size per trial [IQR 13.4 to 50%, range 5.3 to 63.2%], information available for 9 trials)**					
**COVit** (DRKS00021214)	Nicotinamide vs. placebo in outpatients on complete symptom resolution (double-blind)	840	2020-04 to n.r.	U Schleswig Holstein (Kiel)	No
**RECOVER** (EUCTR2020-001632-10-DE)	Convalescent plasma in inpatients on clinical improvement (open-label)	174	2020-08 to n.r.	n.r.	No
**CovidVal01** (DRKS00021732)	Valsacor or Ramipril vs. placebo in inpatients and outpatients on clinical improvement (double-blind)	300	2020-08 to n.r.	H Leipzig (St. Georg)	No
**COMET** (EUCTR2020-001936-86-DE)	Convalescent plasma in inpatients on progression to disease (open-label)	340	2020-08 to n.r.	n.r.	No
**COVID-SMART** (NCT04471636)	Withings ScanWatch ^+^ and 24/7 telemedicine care in outpatients on a combined outcome (hospitalization and unplanned use of emergency medical services) (open-label)	600	2020-09 to 2021-12	U Munich (LMU)	No
**GI-COVID** (NCT04569877)	Molgramostim vs. placebo in inpatients on mechanical ventilation (double-blind)	238	2020-09 to 2022-12	U Giessen, 8 other centers (n.r.)	No
**CVC for COVID-19** (NCT04500418)	Cenicriviroc vs. placebo in inpatients on clinical progression (discharge on day 15) (double-blind)	183	2020-09 to 2021-09	U Berlin (Charité)	No
**COVID-PREVENT** (NCT04416048)	Rivaroxaban in inpatients and outpatients on a combined outcome (venous/arterial thromboembolism, myocardial infarction, non-hemorrhagic stroke, all-cause mortality, intubation, and invasive ventilation) (open-label)	400	2020-11 to 2021-12	U Berlin (Campus CBF), U Berlin (Campus CVK), U Berlin (Campus CCM), U Frankfurt, U Munich (LMU), U Heidelberg, U Dresden, U Wuppertal, U Bochum (Herne), U Schleswig Holstein (Lübeck), U Halle, U Essen, H Berlin (Vivantes Humboldt), H Berlin (Unfallkrankenhaus), H Hennigsdorf (Oberhavel Klinik), H Berlin (Waldfriede), H Bielefeld, H Friedrichshafen, H Berlin (Vivantes Spandau), H Bernau, H Koblenz, H Berlin (Lungenklinik)	No
**HERO-19** (NCT04542408)	Edoxaban vs. placebo in inpatients and outpatients on a combined outcome (all-cause mortality and venous/arterial thromboembolism) (double-blind)	172	2020-11 to 2021-09	U Hamburg, U Duesseldorf, U Hannover, U Munich (TU), U Augsburg, U Freiburg, H Hamburg-Barmbek (Asklepios), H Hamburg-St. Georg (Asklepios), H Hamburg-Altona (Asklepios)	No
**CytoResc** (DRKS00021447)	CytoSorb adsorber in inpatients with an indication for hemodialysis on resolution of vasoplegic shock (open-label)	100	2020-11 to n.r.	U Berlin (Charité), U Ulm, U Duesseldorf, U Erlangen, H Cologne-Merheim, H Passau, H Aschaffenburg	No
**RES-Q-HR** (NCT04681430)	Convalescent plasma or Camostat vs. Camostat placebo or usual care in inpatients and outpatients on clinical status (COVID-19 modified WHO ordinal scale ≥ 4b) (double-blind)	1,094	2021-01 to 2021-11	U Duesseldorf, U Freiburg, U Munich (TU), U Frankfurt, U Essen, H Dortmund	No
**IPCO** (EUCTR2020-002122-82-DE)	Convalescent plasma in inpatients on change in SOFA score (open-label)	58	2021-01 to n.r.	U Erlangen	No
**Trials not yet recruiting (n = 2 trials)**					
**ECMO-VID** (NCT04341285)	ECMO in inpatients on all-cause mortality (open-label)	200	2020-08 to 2022-05	U Tuebingen	No
**NCT04377334**	Mesenchymal stem stromal cells vs. in inpatients on improvement of lung injury score (open-label)	40	2020-10 to 2021-07	U Tuebingen	No
**Terminated trials (n = 4 trials, actually recruited a median proportion of 7.6% of the target sample size per trial [IQR 1.3 to 14.3%, range 0.06 to 16.3%], no missing information)**					
**COMIHY** (NCT04340544)	Hydroxychloroquine vs. placebo in outpatients on duration of symptoms and time to disappearance of the virus (double-blind)	2,700	2020-04 to 2020-10	U Tuebingen	No
**COV-HCQ** (NCT04342221)	Hydroxychloroquine vs. placebo in inpatients on in vivo viral clearance (double-blind)	220	2020-04 to 2021-02	U Tuebingen, U Hamburg-Eppendorf, H Balingen (Zollernalb), H Minden (Johannes Wesling), H Reutlingen (Steinenberg), H Rosenheim (RoMed), H Stuttgart (Robert Bosch)	No
**TOC-COVID** (DRKS00021238)	Tocilizumab vs. placebo in inpatients on ventilator free days (double-blind)	200	2020-05 to 2020-08	U Freiburg, U Bad Krozingen	No
**CYCOV-II** (NCT04385771)	Cytokine adsorption in inpatients requiring veno-venous ECMO on interleukin-6 reduction and time to successful ECMO-explantation (open-label)	80	2020-09 to 2021-10	U Freiburg, U Munich (LMU), U Munich (TU), Saarland (Homburg), H Ibbenbueren, H Ludwigsburg, 3 others (n.r.)	No
**Completed trials (n = 3 trials, actually recruited a median proportion of 100% of the target sample size per trial [IQR 99.5 to 106.7%, range 99.1 to 113%], no missing information)**					
**CYCOV** (NCT04324528)	Cytokine removal therapy in inpatients requiring veno-venous ECMO on interleukin-6 level (open-label)	30	2020-03 to 2021-01	U Freiburg	Peer-reviewed ^45^
**CYTOCOV-19** (NCT04344080)	CytoSorb adsorber in inpatients requiring extracorporeal circulation (continuous renal replacement therapy or ECMO) on significant stabilization of hemodynamics (open-label)	24	2020-04 to 2021-05	U Hamburg-Eppendorf	No
**CAPSID** (EUCTR2020-001310-38-DE)	Convalescent plasma in inpatients on a combined outcome (survival and no longer fulfilling criteria of severe COVID-19) (open-label)	106	2020-08 to 2021-02	U Ulm, U Berlin (Charité), U Marburg, U Frankfurt, U Saarland (Homburg), U Dresden, U Freiburg, U Tuebingen, U Greifswald, U Mannheim, H Karlsruhe, H Stuttgart, H Landshut	Preprint ^46^
**Withdrawn trials (n = 1 trial, never recruited participants)**					
**COVID65plus** (NCT04351516)	Hydroxychloroquine vs. placebo in outpatients on hospitalization and death (double-blind)	0 of 350 (0)	2020-04 to 2020-06	U Tuebingen, U Ulm	No

Abbreviations: ECMO = extracorporeal membrane oxygenation; H = hospital (non-universitary); n = number; n.r. = not reported; SOFA = sequential organ failure assessment; U = university hospital, university medical center, or medical school.

Notes: All trials aimed at treating COVID-19 and had a two-arm parallel group design with no treatment/usual care as control if not stated otherwise. In four trials (CVC for COVID-19, COVID-PREVENT, NIC-002, HERO-19), there was information on commercial co-funding since a private company was named as a collaborator. Two trials (ECMO-VID, NCT04377334) by a principal investigator from a university hospital had an unclear funding type with no information on external commercial or public funding; ^+^ Withings ScanWatch is a smart watch for recording SpO2, ECG, and heart rate; First named trial center is lead/sponsor (exception CAPSID, sponsor: German Red Cross DRK Blood Donor Service Baden-Wurttemberg-Hesse).

From the 38 trials partially conducted in Germany, nine (23.7%) planned to recruit over 1,000 participants compared with five from 27 (18.5%) of the trials that were completely conducted in Germany.

### Actual recruitment in Germany

**Figure 1.**
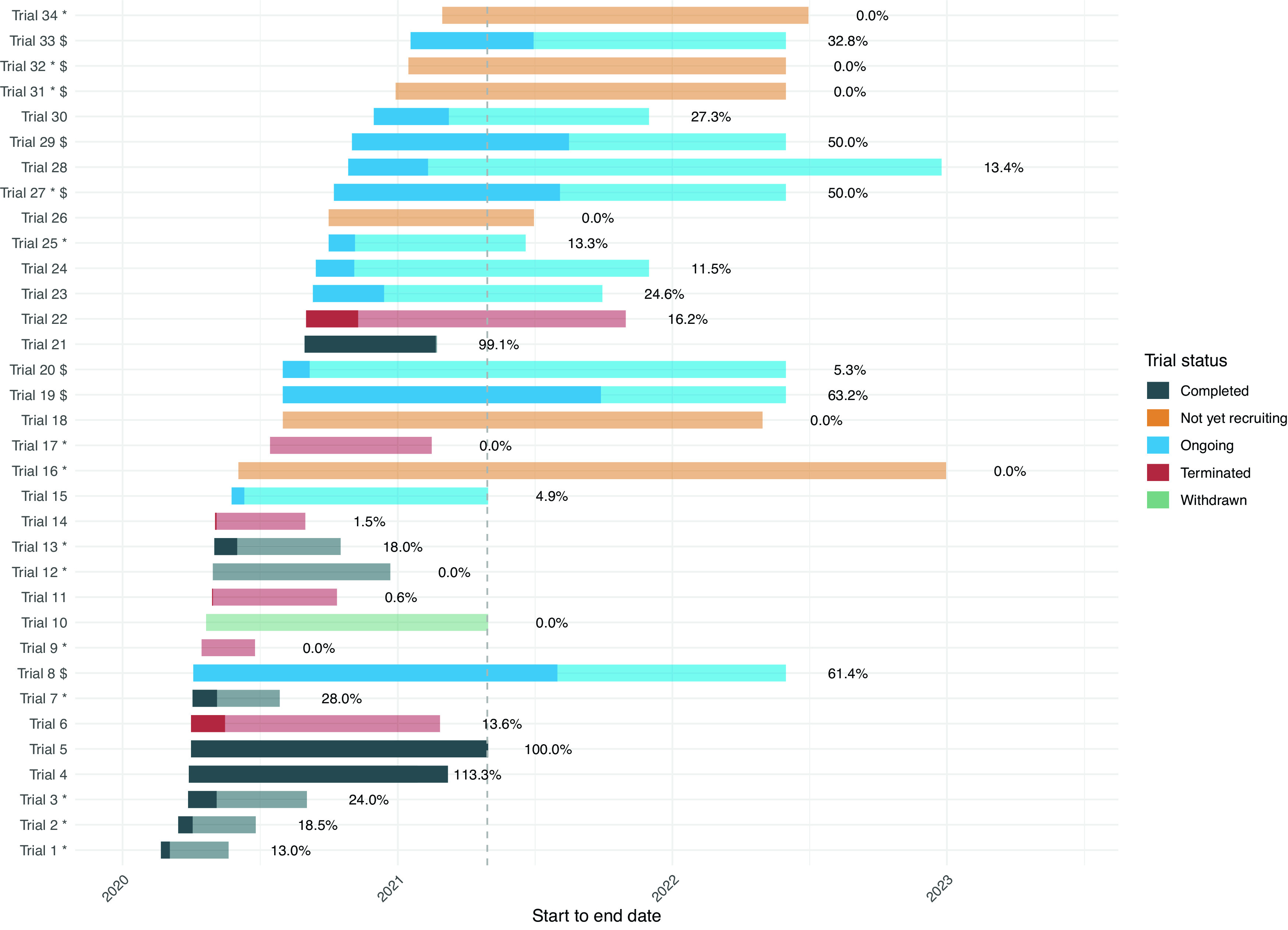
Timeline, status, and actual recruitment for all 34 trials with recruitment information. Notes: The figure illustrates the length of the trials with their start and end date of conduct, sorted by start date and colored by status (based on trial registration details or investigators’ request). The darker part of the bars illustrates the proportion of the target sample size in Germany that was actually recruited within each trial with the corresponding proportion (percentages reported next to the bar). The dashed vertical line corresponds to the timepoint of the recruitment status assessment, April 2021. Trials can be declared as “completed” with or without reaching their target sample size. “Terminated” trials end without reaching their intended goals and started recruiting but are not considered “complete”. “Withdrawn” trials will never start or recruit patients. * Trials that were partially conducted in Germany: the proportion of target sample size focus only on German participants and does not reflect the international recruitment accrual; $ End date was not available and was arbitrarily set at 1 June 2022.

The median number of planned participants from Germany was 106, while the median recruited was actually 15 participants per trial (IQR, 0 to 44), corresponding to a median proportion of 13.4% of the Germany-specific target sample size recruited per trial (IQR, 0 to 29.2%, range 0 to 113%). This proportion was almost identical (13.6%) in the 13 trials that announced a completion date by April 2021 and provided recruitment information ( Table 2). Two of these 13 (15.4%) reached their target sample size (i.e., at least 99%; Figure 1).

From the 65 trials, 11 (16.9%) have not yet been recruiting or, although they had planned to, will never recruit participants in Germany.

The trials with recruitment information from the investigator survey were more often completely conducted in Germany (74.1% vs. 36.8% partially conducted in Germany), more often publicly funded (80% vs. 34.3% industry-funded), and more often registered in the first half of 2020 (64.3% vs. 31.8% second half of 2020; see extended data ^8^).

### Trials identified post-hoc

The search in COVID-evidence in August 2021 identified three additional small trials aiming to include 64 to 130 patients. Two were registered late December 2021, and two have been completed, however none of the results are available. For details see extended data. ^8^

## Discussion

This systematic analysis of the clinical trial research agenda on COVID-19 in Germany showed that from the almost 3,000 trials registered worldwide in 2020, only 65 trials planned to include participants in Germany.

Approximately 20,696 participants from Germany were planned to be included in COVID-19 clinical trials, of which 10,613 patients would be treated for this disease. The typical COVID-19 trial with a German contribution aimed to include 106 persons in Germany, however achieved to recruit a fraction (13.4%, or 15 persons per trial). This estimate did not change when we considered trials which planned to be completed at the timepoint of our recruitment assessment in April 2021. While precise information was not available for all trials, it can be estimated that under 3,000 individuals have been included in COVID-19 RCTs in Germany (13.4% of the 20,696 German participants planned in 55 of 65 trials). In trials for COVID-19 treatments, the estimated total participants were approximately 1,500 (13.5% of 10,613 participants in 48 of 56 treatment trials). This is a small fraction of the approximately 155,000 COVID-19 hospitalizations reported during 2020 in Germany. ^11^ These data indicate that in Germany about 1 out of 100 hospitalized patients participated in a trial to investigate potential COVID-19 therapies, while in the United Kingdom, 1 out of 6 hospitalized patients for COVID-19 took part in the RECOVERY trial. ^12^

It was also unexpected that despite the very prominent and successful position of Germany in research and development of highly effective vaccines, more German participants have not contributed to the overall vaccine research agenda.

There was a considerable public investment in clinical research for COVID-19. The German Federal Ministry of Education and Research (BMBF) spent 1.6 billion € for research projects related to COVID-19, a large part being allocated to vaccine development. ^7^ The RECOVERY trial reported the cost per patient at £250, corresponding to a total of approximately 1 million Euro for a trial with 3,000 persons. ^13^ As illustrated in Figure 1, the planned number of participants in Germany has been reached in three of the completed, and none of the early terminated trials. Moreover, 2 out of the 13 trials that announced a completion date by April 2021 reached their target sample size. The proportions of already recruited participants in the ongoing trials agree with the overall interpretation, and do not exclude delayed recruitment, which is further corroborated by the six not yet recruiting trials. Decisions for early termination may very well indicate reasonable and well-founded strategic decisions to avoid wasting research resources, however it may also reflect recruitment difficulties or other challenges. An overestimation of eligible participants or prejudiced views by recruiters and participants on trial interventions are common reasons for low recruitment. ^14^ However, the massive impact of the COVID-19 pandemic on health care systems and clinical research institutions has challenged trial recruitment as well. ^15, 16^ Most countries do not have the long-established and effective clinical trial infrastructure and academic environments such as the UK, where the feasibility of rapid setup and implementation of massive clinical trials has been impressively demonstrated. The RECOVERY trial was planned in two days, included more than 10,000 patients with the first patient enrolled after nine days, and discovered the first mortality-lowering treatment for COVID-19 in two months. ^2^ The highly pragmatic and embedded-in-usual-care study design were major drivers of this unprecedented successful clinical research.

As the reasons that might have led to successful or unsuccessful recruitment in the German trials were not assessed in this study, further research is needed to understand the recruitment challenges, and how they can be mitigated to facilitate recruitment success for clinical trials in Germany in situations where decision-relevant evidence is urgently needed. A careful comparison of the German research environment for clinical trial research with international circumstances seems warranted.

The focus of the trial research in Germany was similar to the worldwide trial landscape with a strong weight on exploring treatment with drugs and biologicals. ^1^ Our results indicate that there were no RCTs registered in 2020 assessing strategies to control the pandemic spread, social distancing, or behavioral interventions although those have been strictly applied in Germany. There was also no German contribution with RCTs conducted in nursing homes, kindergarten, childcare, or schools although the role of these settings was under considerable discussion. Conversely, there are international examples of RCTs aiming to determine the best strategies for preventing virus transmission. Many trials in the US, for example, were planned to investigate how tailored programs to increase adherence to preventive measures would affect transmission rates. ^17– 19^ Another US randomized trial included almost 20 million people to assess how digital information may affect local case rates. ^20^ Different testing strategies to ensure safe gatherings at mass events have also been investigated in Spain, ^21^ France ^22^ and Norway. ^23^ In case of facing future pandemics with similar challenges, such research would be highly important to develop evidence-based public health interventions and to improve policy-making in Germany.

This study has several limitations. First, our sample depended on accurate trial registrations and reports in the biomedical literature. We cannot exclude those trials conducted in Germany that have not been identified, for example because they were not registered, the registry data gave no indication of a German contribution, or the registry entry was delayed. Nevertheless, searching the Germany-specific registry DRKS did not yield any additional trials. The use of the most recent version of COVID-evidence in August 2021 resulted in just one more trial not previously captured by the filter, and two additional studies registered very late (28 and 30 December 2020) not previously captured due to time lags in registries. While these three studies are not included in the recruitment analysis, they have similar characteristics to the other trials and would not impact our overall interpretation. Second, although we obtained information for more than half of all included trials in an investigator request, we still had incomplete data on the actual recruitment in Germany with imbalances related to some trial characteristics. For example, we had recruitment information for 34.3% of the industry-funded trials compared to 80% of the publicly-funded trials. However, it is unlikely that the other trials have substantially higher recruitment and completion rates, and that missing recruitment information significantly affected our results. Even a five-fold higher recruitment rate in these remaining trials would not change the overall picture: which is that most trials have not been successfully recruited. Finally, the estimation of a target sample size might not be applicable for the few trials with an adaptive design, since sample size (and other methodological trial characteristics) may change over time. Therefore, our analysis related to adaptive designs should be seen contextualized within the trial progress. However, the median reported target sample size for trials with and without adaptive designs was similar.

## Conclusions

The overall German contribution to the worldwide clinical trial research agenda for COVID-19 has been relatively modest. While few excellent examples of successful individual trial recruitment exist, most trials were not able to meet their goals and did not deliver the much needed evidence. A close evaluation and international comparison of the challenges and barriers for conducting clinical trials in Germany is urgently needed.

## Data availability

### Extended data

Open Science Framework (OSF): COVID-evidence: A living database of trials on interventions for COVID-19/Clinical trial research on COVID-19 in Germany – a systematic analysis.

DOI: https://doi.org/10.17605/OSF.IO/CD6RQ. ^8^

The supplement file contains the following extended data:

- Data sources and search strategy
- Email template/Recruitment information request
- Email template/Data confirmation request
- PRISMA flow diagram for trial identification
- Table 1 a. Characteristics of randomized COVID-19 trials planned to be conducted in Germany
- Table 1 b. Trial characteristics and recruitment details
- Characteristics of trials identified post-hoc

Open Science Framework (OSF): COVID-evidence: A living database of trials on interventions for COVID-19.

DOI: https://doi.org/10.17605/OSF.IO/GEHFX. ^9^

The project contains the following extended data:

- COVID-evidence protocol

## Reporting guideline

Open Science Framework (OSF): PRISMA checklist for ‘Clinical trial research on COVID-19 in Germany – a systematic analysis.

DOI: https://doi.org/10.17605/OSF.IO/CD6RQ. ^8^

Data are available under the terms of the Creative Commons Zero “No rights reserved” data waiver ( CC0 1.0 Public domain dedication)

## Author contributions

JH performed conceptualization, formal analysis, investigation, methodology, project administration, validation, visualization, original draft preparation, and review & editing of the article. AR and MB conducted investigation, validation, and review & editing of the article. PD conducted data curation and review & editing of the article. PJ performed conceptualization, formal analysis, investigation, methodology, project administration, supervision, validation, visualization, original draft preparation, and review & editing of the article. LGH was involved in conceptualization, funding acquisition, methodology, project administration, resources, supervision, validation, visualization, original draft preparation, and review & editing of the article.

## Ethical approval

Not required, this article does not contain any personal medical information about any identifiable living individuals.
