# Supplementary material for: Clinical trial research on COVID-19 in Germany – a systematic analysis
Source: F1000Res. 2021 Sep 10;10:913. [Version 1] doi: 10.12688/f1000research.55541.1 (PMC10739183; doi:10.12688/f1000research.55541.1)
Supplement: Peer review history

# Reviewer report: Reviewer response for version 1

**DOI:** 10.5256/f1000research.59126.r179772

**Published:** 2023-12-21

**Authors:** Waldemar Siemens

**Affiliations:** 1 Institute for Evidence in Medicine, Medical Center–University of Freiburg, Faculty of Medicine, University of Freiburg, Freiburg, Germany; 2 Cochrane Germany, Cochrane Germany Foundation, Freiburg, Germany

Thank you for the opportunity to review this analysis on clinical trial research on COVID-19 in Germany for which the authors searched the trial registries and well-known platforms like (L-OVE) and the Cochrane COVID-19 Study Register. The data is shown in a descriptive way and underline the modest contribution of Germany regarding RCTs on COVID-19. I have only some minor comments. The authors could elaborate more on the funding structure and trials infrastructure in Germany to give the reader more context knowledge (e.g., background and/or discussion). I also would encourage the authors to justify in the background why the analysis focused on Germany, why not on Switzerland or another country? In general, I agree with the way the results are shown and the corresponding conclusion. 

**Minor comments:**

 Abstract:

- Please add databases you searched.

Background: 

- Please justify why RCTs in Germany were analyzed.
- You could give more information on the funding structure and trial infrastructure in Germany.

Methods: 

- “Four authors (JH, PJ, AR, MB) extracted data on trial characteristics” – no double extraction, correct?

Results:

- “Most trials were double blinded” - Who was exactly blinded? Had you a closer look or did you just extract if the expression 'double blind' was used?

Table 1:

- Sometimes there is a number after the decimal point and sometimes not. Please be consistent.

Discussion:

- You could give more information on the funding structure and trial infrastructure in Germany to enhance the understanding of the results for the reader.
- Do authors plan to re-assess the data in 2023? (all trials should have finished recruiting then).
- You mention some valid implications for research. Do you also have recommendations or implications for practice? E.g., trialists, funding bodies. Or implications for (meta-)researchers how?

Is the work clearly and accurately presented and does it cite the current literature?

Yes

If applicable, is the statistical analysis and its interpretation appropriate?

Yes

Are all the source data underlying the results available to ensure full reproducibility?

Yes

Is the study design appropriate and is the work technically sound?

Yes

Are the conclusions drawn adequately supported by the results?

Yes

Are sufficient details of methods and analysis provided to allow replication by others?

Yes

Reviewer Expertise:

Meta-research, meta-analysis, GRADE

I confirm that I have read this submission and believe that I have an appropriate level of expertise to confirm that it is of an acceptable scientific standard.

# Reviewer report: Reviewer response for version 1

**DOI:** 10.5256/f1000research.59126.r124218

**Published:** 2022-03-11

**Authors:** Anthony De Soyza

**Affiliations:** 1 Population Health Science Institute, NIHR Newcastle Biomedical Research Centre, Newcastle University, Newcastle, UK; 2 Comprehensive Research Network Respiratory Disorders Group, National Institute for Health Research, Newcastle, UK

This is an interesting and candid report by Hirt, Hemkens and colleagues highlighting limited successes in recruitment into COVID-19 research during the specified time using trial registrations in 2020 in Germany.

 It highlights that recruitment was rarely satisfactory with a median rate of recruitment vs target of ~13%. The median number of planned participants from Germany was 106, while the median recruited was actually 15 participants per trial (IQR, 0 to 44). The manuscript displays data on the recruitment rates of studies identified by multiple methods and covers a broad range of COVID facing approaches including devices (ECMO), anti virals and anti- inflammatory therapies.

 The work has many commendable features but there are some limitations that could be more fully discussed.

 Firstly the registration period may not relate to actual study opening. It is therefore unclear how many studies were impacted by either slow start up and/or rapid closure. The hydroxychloroquine studies noted, for example, may well be explained by the rapid success of RECOVERY reporting lack of benefit meaning early closure. These data may be included within the manuscript but are not easy to identify - some of the explanatory text is more identifiable in this matter in the discussion.

 The poor recruitment and attempts to understand why is important as solutions to resolving poor recruitment within a pandemic are complex. The UK experience note din the discussion had a number of factors contributing to “success” ranging from “infrastructure “commented upon by the authors. This relates to an expectation research is part of normal clinical practice and there is specific funding into most organisations to deliver upon this via in England the National Institute for Health Research (NIHR) Comprehensive research network (CRN).

 What is not well described in this paper is the differences in approvals/ site selection/ national imperatives and process for dealing with competing studies in Germany. The trials infrastructure in Germany is not discussed (who decides which sites take on which studies  and what resource if any is funded nationally? What is the incentive to do pragmatic non-commercial studies vs fee paying low volume complex industry studies? One cannot therefore develop an understanding of why there were such differences in experiences within Germany (some studies did hit targets as reported herein) and why there are differences between countries.

 These all need to be discussed/ dissected to understand how coordination of research efforts was/ was not a root cause of the poor recruitment as opposed to lack of staff capacity/selecting small volume high intensity trials vs low intensity pragmatic trials.

 The authors may wish to clarify the tables/ figures where figure 1 notes trials 1-34 but does not directly link them to the preceding tables. It would be useful to understand why some of the studies hitting target were different to those not.

 Other issues relate to ongoing open studies that are limited to Germany only - there can be investigator led reticence to close down failing studies – if these studies are still failing to recruit and are closed down then recruitment success to studies overall will fall further as a % of studies hitting target. This limitation should be noted

 The authors may wish to note Cunliffe *et al *(2020) How achievable are COVID-19 clinical trial recruitment targets? A UK observational cohort study and trials registry analysis - PubMed (nih.gov) an analysis of clinical trials feasibility in the UK showing that there was likely an over commitment within the UK that needed national coordination to prioritise the most important studies. ^1^

 The UK National Institute for Health research (NIHR) developed and rapidly implemented an Urgent Public health (UPH) Panel. It pulled in very broad expertise and linked with governmental advisory panels from therapeutics and vaccines plus trials infrastructure delivery & methodology experts to help focus efforts on selecting and then delivering studies. This huge body of work involved reviewing 1000s of studies thrice weekly for much of 2020 to drill down a focus; 95 were listed of which the initial 5 included RECOVERY and PRINCIPLE studies (listed from Jan 2020-Mach 2020). See Urgent Public Health COVID-19 Studies | NIHR.

 There are very many challenges in delivering research successfully even more so in the middle of a pandemic. The authors should be commended on writing the first chapter in Germanys’ book on this. The authors identify the success was below ideal. The challenges are now to identify where improvements can be made to ensure in the future, Germany can harness the potential to deliver research more rapidly.

Is the work clearly and accurately presented and does it cite the current literature?

Partly

If applicable, is the statistical analysis and its interpretation appropriate?

Partly

Are all the source data underlying the results available to ensure full reproducibility?

Partly

Is the study design appropriate and is the work technically sound?

Partly

Are the conclusions drawn adequately supported by the results?

Partly

Are sufficient details of methods and analysis provided to allow replication by others?

Yes

Reviewer Expertise:

Research background Respiratory medicine, Clinical Trials, COVID 19,

I confirm that I have read this submission and believe that I have an appropriate level of expertise to confirm that it is of an acceptable scientific standard.

## References

[1] : How achievable are COVID-19 clinical trial recruitment targets? A UK observational cohort study and trials registry analysis. *BMJ Open* .2020;10(10) : 10.1136/bmjopen-2020-044566 10.1136/bmjopen-2020-044566 PMC753663433020111
